# Clinical, functional and inflammatory evaluation in asthmatic patients after a simple short-term educational program: a randomized trial

**DOI:** 10.1038/s41598-021-97846-8

**Published:** 2021-09-14

**Authors:** Soraia Nogueira Felix, Rosana Câmara Agondi, Marcelo Vivolo Aun, Clarice Rosa Olivo, Francine Maria de Almeida, Thais Santos Amorim, Julia Caroline Cezario, Pedro Giavina-Bianchi, Iolanda de Fátima Lopes Calvo Tiberio, Milton de Arruda de Martins, Beatriz Mangueira Saraiva Romanholo

**Affiliations:** 1 Instituto de Assistência Médica Ao Servidor Público Estadual (IAMSPE), Sao Paulo, SP Brazil; 2 Serviço de Imunologia Clínica e Alergia, HCFMUSP, Universidade de São Paulo, Sao Paulo, SP Brazil; 3 Universidade Cidade de Sao Paulo (UNICID), Sao Paulo, SP Brazil; 4 Laboratório de Terapêutica Experimental (LIM-20), Faculdade de Medicina da USP (FMUSP), Universidade de Sao Paulo, Sao Paulo, SP Brazil

**Keywords:** Diseases, Health care, Medical research, Signs and symptoms

## Abstract

This study aimed to evaluate the clinical evolution, functional parameters and inflammatory activity of asthma in patients who submitted to an educational intervention. 58 adult patients over 18 years of age with partly controlled and uncontrolled asthma were randomized into an intervention group (IG) (N = 32) and a control group (CG) (N = 26) and evaluated for 12 weeks. The Asthma Control Test (ACT), Asthma Control Questionnaire (ACQ), Asthma Quality Life Questionnaire (AQLQ) and Beck Depression Inventory (BDI) questionnaires were applied. Spirometry, exhaled nitric oxide (NO), exhaled breath condensate (EBC) and induced sputum (IS), measurement of the peak flow and symptoms were performed. The IG patients received an educational activity for 30 min applied by a nurse. Statistical analysis: analysis of variance with repeated intragroup measures. IG presented a decreased number of eosinophils in IS and IL-17A in EBC, an increase in the percentage of FEV_1_ before and after bronchodilator and an improvement in quality of life compared to the CG. There was an improvement in depression levels and a decrease in IL-4 and IL-5 in the IS and in the EBC in both groups. Our results suggest that an educational intervention can bring benefits concerning the control of inflammation, lung function alterations, quality of life and levels of depression in asthmatic patients. Registration: ClinicalTrials.gov; NCT03655392.

## Introduction

It is estimated that approximately 358 million individuals worldwide are affected by asthma^1^, and the global prevalence can vary from 1 to 18%^2^. Chronic inflammation in asthma is a consequence of the participation of several mediators that lead to influx of inflammatory cells and airway remodeling as a consequence of goblet cell metaplasia, excessive subepithelial collagen deposition, airway smooth muscle hyperplasia, and increased vascularity. These characteristic features are orchestrated mainly by Type 2 (Th2) cells and cytokines^3–5^.

Thus, the main goal of asthma treatment is to reduce inflammation in the airways and consequently control the disease and its symptoms. Asthma treatment includes inhaled corticosteroids (ICS) and long-acting bronchodilators (LABA), and, more recently, for severe asthma, biologicals that target Th2 mediators. Factors contributing to an uncontrolled asthma are poor adherence and inappropriate inhaler technique. Studies have found that most patients do not use their inhalation device correctly^2,6–8^. The correct use of these drugs is associated with better efficacy and fewer collateral effects related to therapy^9^.

Inhaled corticosteroids are the basis of treatment for most cases of asthma. Their constant use is associated with an improvement of symptoms and a reduction of morbidity and mortality related to disease^2,10,11^.

Several studies showed that poor adherence to asthma medications and inadequate inhaler technique were associated to difficult-to-control asthma. The asthma education program is considered one of the fundamental pillars for adherence and proper treatment of the disease. It is estimated that more than half of patients treated for asthma do not have adequate adherence to the prescribed medication or do not perform the treatment properly^2,12,13^.

A review of interventions for inhaler techniques concluded that most of the studies showed that an intervention improved inhaler technique when assessed by a checklist or dichotomously; and that it was true for both children and adults with asthma^14^. Melani et al. observed that critical errors in the inhalation technique were observed in 36% of the patients with chronic airflow obstruction^15^.

We believe that an educational intervention aims at better comprehension on asthma care, including environmental control, adherence to medication use, and correct use of the inhalation device. These interventions can improve clinical, functional and inflammatory parameters of asthma. Thus, the objectives of this study were to assess asthma control, quality of life, levels of depression, lung function and inflammatory parameters of patients with asthma before and after an educational intervention.

## Methods

### Study design

This study was a prospective, randomized, controlled trial approved by the Hospital Research Ethics Committee of the University of Sao Paulo (protocol number: 11496/14); the recruitment and protocol was carried out between 2015/2017. It was registered at ClinicalTrials.gov, registration number NCT 03,655,392, first registered in 03/28/2018 (retrospectively recorded) (https://clinicaltrials.gov/ct2/show/NCT03655392).

Individuals with a clinical asthma diagnosis were recruited from an outpatient allergy and immunology clinic at a university hospital. Written informed consent was obtained from all participants included in the study. All participants received explanations, signed and received a copy of the written consent form. Participation was voluntary and participants were free to withdraw from the study at any stage. Participants were recruited between July 2015 and December 2017.

### Eligibility criteria

Adults between 18 and 69 years of age with clinical diagnosis of asthma^2^; asthma with partly controlled or uncontrolled symptoms^2^; continuous medical follow-up and patients using ICS with or without LABA (long-acting beta-agonist) for at least 1 year; increase in FEV_1_ of > 12% and > 200 ml from baseline 10–15 min after 400 mcg Salbutamol; dose of ICS stable in the last 8 weeks before recruitment; and nonsmokers or ex-smokers less than or equal to 10 pack-years. Subject characteristics are summarized in Table 1.

**Table 1:** Baseline data.

	IG	CG	Total	p
	Number (%) or mean ± SD			
**Gender**				
Female	23 (72)	17 (65)	40 (69)	0.806
Male	9 (28)	9 (35)	18 (31)	
Total	32 (100)	26 (100)	58 (100)	
**Age** (mean ± SD)	55.06 ± 11.29	51.61 ± 14.16		0.420
**Education (years)**				
Illiterate—0	3 (9)	2 (8)	5 (9)	0.408
≤ 8	13 (41)	13 (50)	26 (45)	
≤ 12	12 (38)	5 (19)	17 (29)	
> 12	4 (12)	6 (23)	10 (17)	
Total	32 (100)	26 (100)	58 (100)	
**Treatment**				
Formoterol + budesonide (capsules)	23 (72)	21 (80)	44 (76)	0.350
Salmeterol + Fluticasone (diskhaler spray)	2 (6)	2 (8)	4 (7)	
Formoterol + budesonide liquid (spray)	7 (22)	2 (8)	9 (15)	
Beclometasone (spray)	0	1(4)	1 (2)	
**Onset of asthma**				
Childhood	14 (44%)	11 (42)	25 (43)	0.876
Adult	18 (56)	15 (58)	33 (57)	
**Level of asthma symptom control**				
Well controlled	0	0	0	
Partly controlled	8 (25)	14 (54)	22 (38)	0.048*
Uncontrolled	24 (75)	12 (46)	36 (62)	
Total	32 (100)	26 (100)	58 (100)	
Endotype (induced sputum)**				
Paucigranulocytic	21 (66)	19 (73)	40 (69)	0.027*
Neutrophilic	3 (9)	7 (27)	10 (17)	
Eosinophilic	6 (19)	0	6 (10)	
Mixed	2 (6)	0	2 (4)	
**Former smoking**				
> 5–10 years	0	4 (15)	4	0.087
> 10 years	10 (31)	6 (23)	16	

Data are expressed as the numbers and percentages—N (%) or means ± SD (standard deviation). Statistical analysis: Chi-square (qualitative variables) and T-test (numerical variables).

*CG* control group, *IG* intervention group. T1 = day 0 (baseline). T2 = day 28. T3 = day 56.

*p < 0.05 compared to T1. **Values are expressed as the percentages of patients with sputum samples sufficient for analysis. Eosinophilic: eosinophils > 2.5%, neutrophils ≤ 54%; neutrophilic: eosinophils ≤ 2.5%, neutrophils > 54%; mixed: eosinophils > 2.5%, neutrophils > 54%; paucigranulocytic: eosinophils ≤ 2.5%, neutrophils ≤ 54%. IG = intervention group. CG = control group.

Exclusion criteria: clinical asthma diagnosis and upper respiratory tract infection less than 30 days before; systemic steroids within 4 weeks of enrollment; pregnancy; other lung or uncontrolled chronic disease and chronic obstructive pulmonary disease (COPD) according to the Global Initiative for COPD (GOLD)^16^.

### Outcome measures

**Figure 1:**
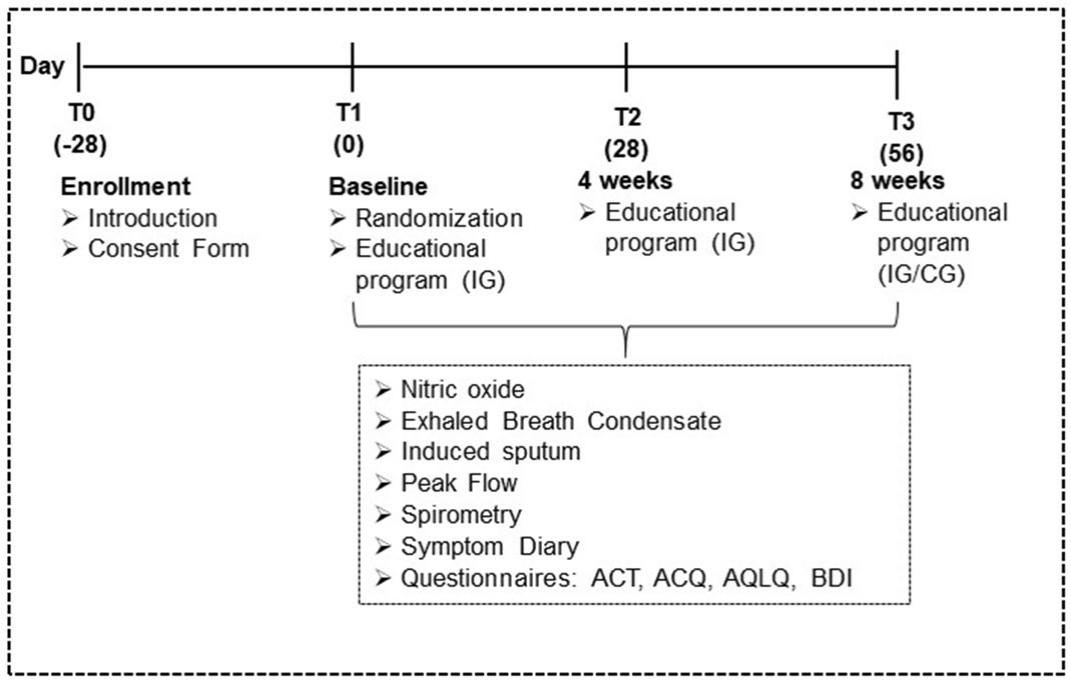
Study design**.** * Phone calls were made to the IG group every two weeks during the study to remind them about the correct use of medication and to remember the date of the study visit.

Individuals were recruited after regular medical visits in outpatient clinics, by the researchers and physicians, after routine outpatient medical care. The study protocol was explained to the possible participant, and signed informed consent was requested within 28 days (T0). Subjects were followed for 56 days at three visits every 4 weeks (day 0 = T1 (baseline), day 28 = T2 and day 56 = T3) (Fig. 1).

At the first visit (T1), the patients were randomized in the order of inclusion in a 1:1 ratio for the IG (intervention group) or CG (control group) groups. The symptom diary and a manual and portable peak flow meter (Mini-Wright, Clement Clark International) were delivered to all patients. Then, they answered the following questionnaires administered by a blind investigator: Asthma Control Test (ACT)^17^, Asthma Control Questionnaire^18^, Asthma Quality Life Questionnaire (AQLQ)^19^ and Beck Depression Inventory II (BDI II)^20^. After, individuals underwent spirometry for the collection of exhaled breath condensate (EBC) and induced sputum (IS). All these data were collected from both groups at the three study visits (T1, T2 and T3).

Additionally, IGs were submitted to educational intervention at all three visits (T1, T2 and T3) and received phone calls every 2 weeks, reinforcing the educational content and the date of return. CGs were only advised as to the date of return and received phone calls 2 days before to confirm attendance.

Educational intervention was applied by a trained nurse in a 30-min session. Its main objective was to verify whether the patient was using the ICS properly and, if necessary, to teach the correct inhalation technique. It included the asthma education components recommended in the Global Initiative for Asthma (GINA) main report^2^ and a simplified explanation of this disease and its main characteristics, guidance on inhalation technique using illustrations, a script and an explanatory leaflet.

Skills for correct medication inhalation from a metered-dose inhaler and for peak flow measurement were taught and practiced. The inhalation technique was explained and demonstrated to the patients. As a complementary material, a four-minute explanatory video addressing the correct inhalation technique was shown (available at http://www.incor.usp.br/sites/incor2013/videos/asma-dpoc/). Environmental control was also briefly addressed, and IG subjects were guided on measures to avoid exposure to allergens and irritants.

Finally, patients were invited to demonstrate the inhalation technique. When necessary, the technical errors were corrected by the nurse until the inhalation technique was performed correctly. This activity was performed considering the particularities of each patient.

The symptoms of asthma and peak flow measurement were recorded in a diary of symptoms already used by patients in hospital outpatient clinics^21^. Patients registered these data twice a day in the morning and evening before using ICS. Symptom-free days were accounted for by each asthma symptom individually (cough, wheezing, shortness of breath, waking up at night for asthma and use of rescue medication). At each new visit (T2 and T3), patients returned the symptom diary and the annotation of the peak flow values.

### Clinical evaluation

To access the level of asthma control, the ACT questionnaire scores were used^17^. To evaluate clinical parameters, the ACQ-7 was applied^21^ considering the week before patients fill it out.

Asthma quality of life was assessed using the AQLQ; a higher score indicates a better quality of life^22^. A minimal clinically important difference (MCID) of 0.5 was used. The MCID indicates the minimal difference in mean scores that is regarded as important^23^.

The BDI was used to assess depression levels, it’s a scale that has been used to evaluate related symptoms of depression in the last 4 weeks. The higher the score, the worse the severity of the symptoms^20,24^.

### Functional evaluation

Spirometry was also performed at the three study visits and was analyzed according to the acceptability and reproducibility criteria recommended by ATS/ERS (2005)^25^; a Koko spirometer (N Spire Health, Inc; Longmont, CO, USA) was utilized.

Peak expiratory flow (PEF) measurement was performed after NO (nitric oxide) collection and after each inhalation. The patients were also instructed to perform this measurement three times, twice a day, morning and evening, before the use of the ICS, and to note down the three measurements in the symptom’s diary.

### Inflammatory parameters

Sputum was induced by standard methodology^26,27^. Patients were asked to inhale 400 μg Salbutamol via a metered-dose inhaler 20 min before induction and then inhaled hypertonic saline (3% NaCl) for 7 min; peak flow and symptoms were evaluated before and after each inhalation. A Devilbiss Ultraneb 99 (Devilbiss Corp., Somerset, PA, USA) ultrasonic nebulizer was used.

The measurement of FeNO (fractional exhaled nitric oxide) was performed by chemiluminescence (Sievers 280) according to the recommendations of the ATS^28^. The patients were instructed to blow into a Mylar balloon with an expiratory pressure supported in 12 cm H2O and a stable flow of 200 ml/s. This procedure and analysis were performed by a blinded investigator.

EBC (exhaled breath condensate) was collected for 15–20 min in the current volume to measure cytokines^29^. A condenser (Turbo DECCS System, Medivac SRL, Italy) cooled to − 20 °C for at least thirty minutes was used. The individuals were instructed to perform oral breathing using the mouthpiece of the equipment and with the aid of a nasal clip.

The levels of interleukin (IL)-4, IL-5 and IL-17A were quantified in IS supernatant and EBCs. ELISA (enzyme-linked immunosorbent assay) (EL Human ISA Max Deluxe, Biolegend, San Diego, CA)^30^ was utilized. The tests were performed according to the manufacturer’s guidelines; the samples were analyzed in an ELISA reader (Polaris Model, Celer Biotechnology SA, Brazil).

### Statistical analysis

Descriptive analysis was performed on all patient data (means, standard deviations and medians). Differences between groups were analyzed using Student’s t test, chi-square and one-way repeated measures analysis of variance (ANOVA). Intragroup analysis was compared by means of repeated measurement ANOVA. The confidence interval was 95% (p < 0.05). A statistical package was used for tests (Sigma Stat 3.11, San José, CA, USA)—descriptive analysis, differences between groups and intragroup, sample calculation) and SPSS 20.0 (odds ratio (OR) and chi-square). To calculate the odds ratio, the rates of change were compared between the groups (IG/CG) at different times of assessment: T1–T2, T2–T3, T1–T3 (95% confidence interval).

Sample size estimates were based on the estimated effect of the intervention on eosinophils in induced sputum according to a previous study^31^. The sample size calculation took into account a minimum mean difference of 67% for eosinophils, with an expected difference in means of 8 and a standard deviation of 10, test power of 80% and alpha of 0.05, thus resulting in a requirement of 13 patients in each group.

### Ethics approval

This study was approved by the HCFMUSP Research Ethics Committee, protocol number 639.895 in accordance with the ethical standards as laid down in the 1964 Declaration of Helsinki. The study was registered and approved according to the country’s standards on the Brazil Platform (*Plataforma Brasil*), number CAEE 25319213.5.0000.0068 (http://plataformabrasil.saude.gov.br/login.jsf).

### Consent to participate

Informed consent was obtained from all individual participants included in the study. All participants received explanations, signed and received a copy of the written consent form.

### Consent for publication

The authors affirm that human research participants provided informed consent for publication of the study data without personal identification. In the written consent form, all participants authorized the publication of the data for this study, without any individual identification related to the participants. The confidentiality and privacy of any personal data were respected in this study.

### Conference presentation

Part of this work was presented at the International Conference of the American Thoracic Society in San Diego, California, 2018.

## Results

### Patient distribution

**Figure 2:**
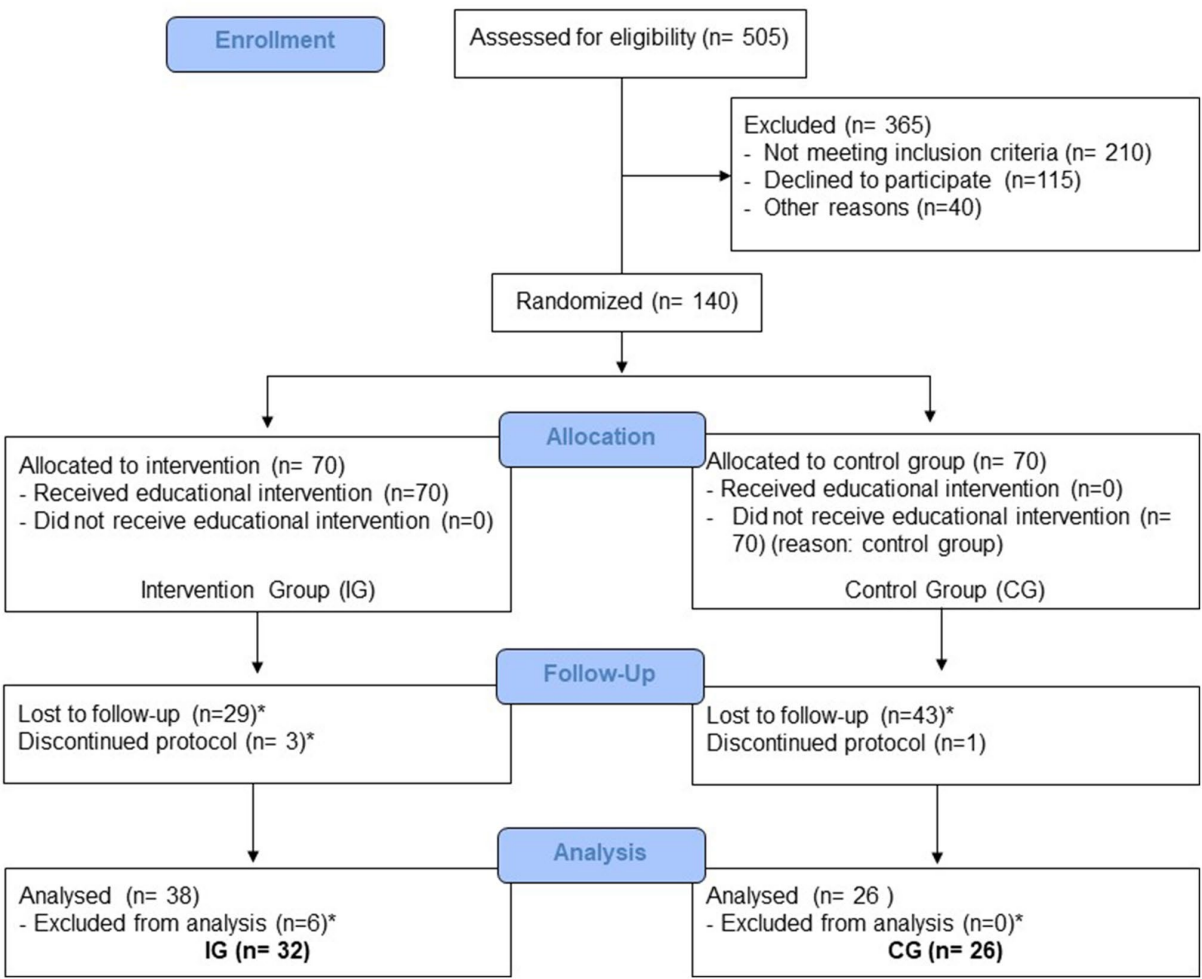
Enrollment (CONSORT diagram, 2010).

A total of 505 individuals were assessed for eligibility and 365 were excluded, 210 for not meeting the criteria, 115 declined to participate and 40 for other reasons. 140 subjects were distributed equally and randomly into the groups: IG (n = 70) and CG (n = 70). During the protocol, 76 patients lost follow-up, 29 from IG and 44 from CG. The claimed reasons for that were: lack of time to participate, other health treatments not related to this study, difficulties to get into hospital, change of address or employment, difficulty in missing work for treatment, among other reasons. Three patients were removed from IG because they presented a slight respiratory discomfort after inhalation with hypertonic saline solution. One patient was removed from CG for emotional disorder. After completing the protocol, we excluded 6 patients from IG for insufficient samples. At the end, 58 patients completed the entire study (IG n = 32) (CG n = 26) (Fig. 2).

### Clinical parameters

**Figure 3:**
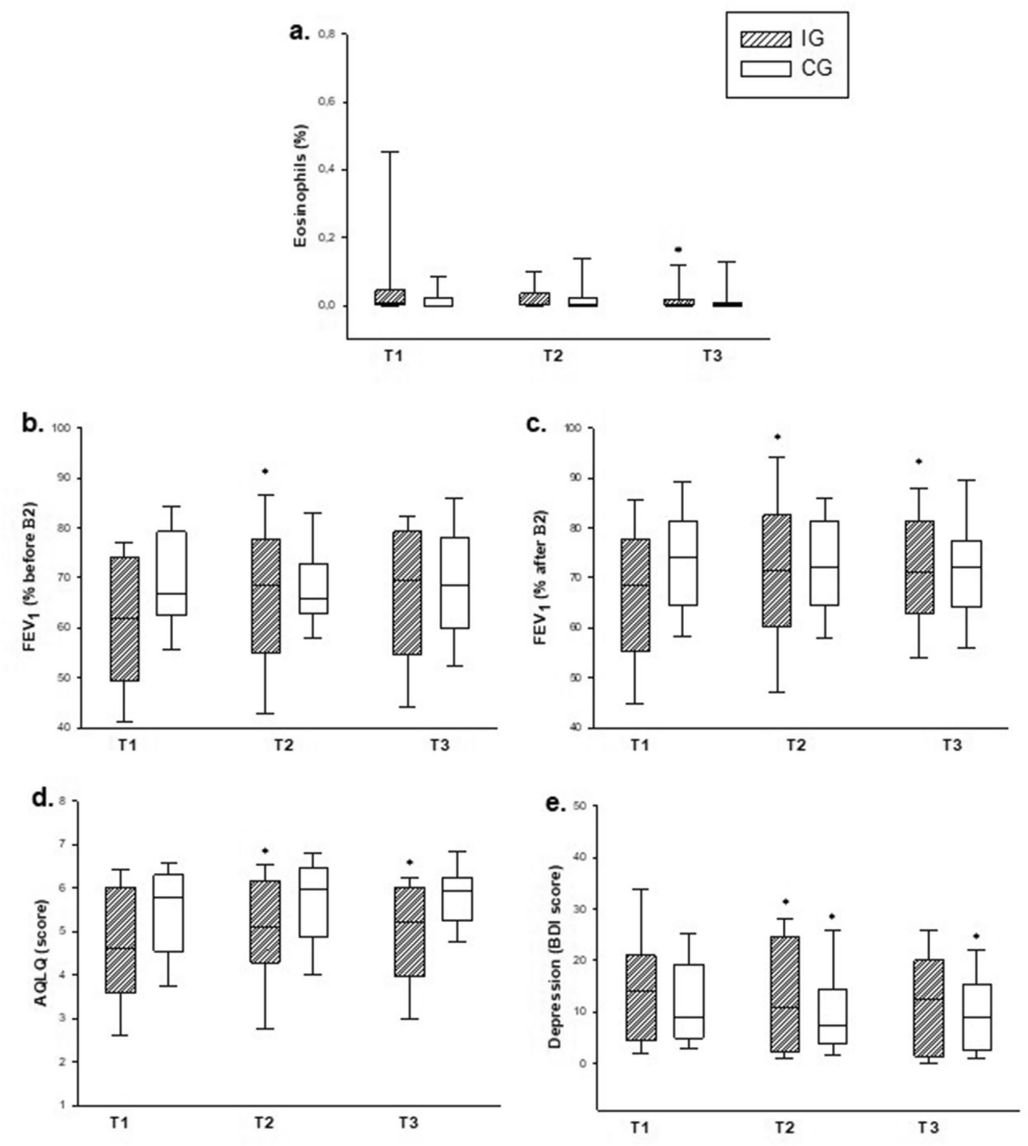
Data were compared in the three evaluations (T1, T2 and T3). IG = intervention group. CG = control group. T1 = day 0 (baseline). T2 = day 28. T3 = day 56. FEV_1_ = Forced expiratory volume in one second. AQLQ = Asthma Quality Life Questionnaire. BDI = Beck Depression Inventory. (**a**) Induced sputum eosinophils (%). (**b**) FEV_1_ before β2 (%). (**c**) FEV_1_ before β2 (%). (**d**) Quality of life (AQLQ score). (**e**) Depression levels (BDI score). *Compared to T1. The boxes represent the 25th to 75th percentiles (SD), the lines within the boxes represent the median values, and the bars represent the 10th and 90th percentiles. Statistical analysis: One-way ANOVA (intragroup analysis).

In evaluation of quality of life (AQLQ), we found an improvement in IG patients when comparing T1 with T2 or T3 moments (Fig. 3d, Table 2) (*p = 0.005). There were no differences between T1, T2 or T3 in CG.

**Table 2:** Clinical parameters measured in CG (control group) and IG (intervention group) in T1 (day 0, baseline), T2 (day 28 and T3 (day 56).

	Group	T1	T2	T3	p
		Median (25–75%)			
**A. Questionnaires**					
AQLQ	IG	4.68 (3.68–6.01)	5.20 (4.34–6.34)*	5.26 (4.01–6.03)*	0.005*
	CG	5.77 (4.59–6.28)	5.96 (4.87–6.46)	5.93 (5.25–6.21)	0.446
BDI	IG	14.00 (4.68–20.00)	11.00 (2.50–24.00)*	12.50 (2.00–20.00)*	0.002*
	CG	9.00 (5.0–19.00)	7.50 (4.0–14.00)	9.00 (3.0–15.00)*	0.006*
ACT	IG	19.00 (16.00–21.50)	19.50 (17.00–22.00)	21.50 (16.00–24.00)	0.277
	CG	22.00 (20.00–24.00)	23.00 (20.00–23.00)	22.00 (18.00–24.00)	0.632
ACQ	IG	1.42 (1.03–2.14)	1.28 (0.57–2.00)	1.14 (0.57–1.85)	0.269
	CG	1.14 (0.71–1.28)	0.92 (0.71–1.57)	1.06 (0.57–1.71)	0.957
B. Free-symptom days**					
Cough	IG		28.50 (10.50–50.50)	30.50 (19.50–58.00)	0.173
	CG		29.50 (5.00–49.00)	42.50 (6.00–51.00)*	0.028*
Wheezing	IG		31.50 (15.00–53.0)	38.00 (21.50–58.50)	0.081
	CG		40.00 (14.00–51.0)	44.50 (22.00–54.00)	0.337
Dyspnea	IG		34.00 (15.50–53.00)	38.50 (21.50–59.00)	0.384
	CG		41.00 (16.00–51.00)	42.00 (22.00–53.00)	0.348
Wake up (asthma)	IG		36.50 (17.50–56.00)	40.50 (23.50–59.00)	0.202
	CG		39.50 (15.00–52.00)	42.50 (22.00–55.00)	0.377
Emergency (SABA)	IG		37.00 (16.50–54.50)	41.00 (24.00–62.00)	0.139
	CG		41.00 (20.00–51.00)	45.50 (22.00–56.00)	0.256
Peak flow	IG		309.32 (244.88–377.42)	316.15 (271.16–367.21)	0.182
	CG		315.68 (287.11–385.24)	328.23 (293.57–390.00)	0.434
**C. Lung function (%)**					
FEV_1_ before β2	IG	62.00 (49.50–74.00)	68.50 (56.00–77.50)*	69.50 (55.50–78.50)	0.009*
	CG	67.00 (63.00–79.00)	66.00 (63.00–71.00)	68.50 (60.00–78.00)	0.847
FEV_1_ after β2	IG	68.50 (55.50–77.50)	71.50 (60.50–82.00)*	71.00 (64.00–81.00)*	0.004*
	CG	74.00 (65.00–81.00)	72.00 (65.00–81.00)	72.00 (65.00–77.00)	0.500
FEV_1_/FVC before β2	IG	85.50 (77.50–95.00)	84.00 (78.00–92.00)	85.00 (79.50–92.00)	0.499
	CG	90.50 (81.00–101.00)	92.50 (81.00–98.00)	89.00 (81.00–99.00)	0.528
FEV_1_/FVC after β2	IG	85.00 (79.50–92.00)	88.00 (80.00–97.00)	88.00 (80.00–95.00)	0.991
	CG	90.50 (82.00–99.00)	92.50 (81.00–97.00)	92.00 (84.00–99.00)	0.189

(A) Questionnaires; (B) Free-symptom days and (C) Lung function (%). The data are expressed as the medians and percentiles 25–75.

*SABA* short action beta-agonist, *AQLQ* Asthma Quality Life Questionnaire, *BDI* Beck Depression Inventory, *ACT* asthma control test, *ACQ* Asthma Control Questionnaire, *FEV*_*1*_ Forced Expiratory Volume in One Second, *FVC* forced vital capacity, *β2* beta2-adrenergic (bronchodilator).

*p < 0.05 compared to T1. **Symptom Diary was delivered to patients on day 0 and day 28 and returned on day 56. One-way ANOVA.

Regarding depression levels, IG patients presented improvement at T2 and T3 compared to T1 (*p = 0.002). In the CG, we found improvements only in T3 compared to T1 (*p = 0.006) (Fig. 3e, Table 2).

In relation to asthma control when the ACT questionnaire was applied, although IG patients presented an increase of 2.5 points, no difference was observed. Also, in the ACQ analysis, no significant differences were found in symptom-free days or daily measurement of peak flow (Table 2).

In addition, we evaluated the number of days when patients were free of the following factors: cough, wheezing, dyspnea, awakening because of asthma symptoms, rescue medication (SABA) and peak flow. In CG, patients reported more days free of cough at T3 when compared to T2 (*p = 0.028) (Table 2). There were no differences in other parameters.

### Functional evaluation

IG presented an increase in FEV1 before (p = 0.009) and after bronchodilator (p = 0.004) in T2 and T3 when compared to T1 CG (Fig. 3b, c, Table 2). There was no difference between FEV_1_/FVC before and after bronchodilator in IG or CG (Table 2).

### Inflammatory parameters

Both groups presented similar patient characteristics at baseline (T1). According to cellularity in endotype (induced sputum), most patients had paucigranulocytic asthma (69%) compared to neutrophilic, eosinophilic and mixed endotypes (p = 0.027*) (Table 1).

In comparison of differential cell count in sputum, a significant decrease in the eosinophil number was observed at T3 (2 months) compared to T1 in the IG (*p = 0.034) (Fig. 3a, Table 3). We did not find difference in number of neutrophils, lymphocytes and macrophages in both groups at three moments as well as in NO measurement (Table 3).

**Table 3:** Inflammatory profile. The data are expressed as the medians and percentiles 25–75 (SD).

Outcome	Group	T1	T2	T3	p
		Median (25–75%)			
**A. Differential count of cells (%)**					
Eosinophils	IG	0.60 (0.20–3.00)	0.20 (0.00–0.65)	0.20 (0.00–0.85)*	0.034*
	CG	0.00 (0.00–0.20)	0.10 (0.00–0.60)	0.20 (0.00–0.20)	0.568
Neutrophils	IG	35.40 (10.72–56.10)	31.80 (13.55–57.65)	40.60 (6.60–62.85)	0.971
	CG	17.30 (4.40–65.20)	26.60 (2.282–54.40)	14.70 (4.20–47.00)	0.651
Lymphocytes	IG	1.20 (0.800–2.90)	1.40 (0.80–2.05)	1.60 (0.75–2.90)	0.320
	CG	1.00 (0.668–1.40)	1.00 (0.40–1.60)	0.80 (0.60–1.40)	0.911
Macrophages	IG	54.80 (36.10–87.02)	60.20 (40.00–82.45)	56.40 (35.15–90.55)	0.986
	CG	80.40 (32.60–94.60)	72.30 (44.80–96.88)	82.90(49.80–95.20)	0.525
**B. Exhaled nitric oxide (NOEX) (PPB)**					
	IG	24.6 (17.3–34.1)	26.7 (23.0–36.1)	25.5 (15.4–40.3)	0.585
	CG	26.2 (17.8–39.2)	21.0 (17.4–23.0)	22.5 (17.6–30.8)	0.194
**C. Cytokines—induced sputum supernatant**					
IL-4	IG	1.371 (1.224–1.594)	0.970 (0.680–1.124)*	0.858 (0.501–1.092)*	< 0.001*
	CG	1.140 (0.568–1.464)	0.786 (0.671–0.962)	0.419 (0.300–0.590)***	0.001*
IL-5	IG	2.965 (0.967–3.101)	2.428 (1.918–3,126)	1.039 (0.967–1.334) ***	0.010*
	CG	1.840 (1.195–3.200)	3.398 (1.627–4.815)	0.600 (0.380–0.810)***	< 0.001*
**D. EBC cytokines**					
IL-4	IG	0.928(0.800–1.254)	0.730(0.456–0.982)*	0.858(0.450–1.076)*	< 0.001*
	CG	1.124(0.906–1.376)	0.691(0.454–1.147)	0.432(0.388–0,695*	0.011*
IL-5	IG	2.529(1.709–2.903)	1.415(1.306–1.735)*	0.733(0.535–1.025)*	< 0.001*
	CG	3.324(1.937–4.380)	1.223(0.841–1.381)	0.535(0.433–0.765) )***	< 0.001*
IL-17A	IG	0.855(0.633–1.054)	0.832(0.681–1.129)	0.705(0.268–.0.871)**	0.028*
	CG	0.448(0.296–1.181)	0.761(0.665–1.009)	0.583 (0.444–0.661)	0.091

*CG* control group, *IG* intervention group, *T1* day 0 (baseline), *T2* day 28, *T3* day 56, *NOex* Nitric Oxide exhaled, *ppb* parts per billion, *EBC* exhaled breath condensate, *IG* intervention group, *CG* control group.

*p < 0.05. One-way ANOVA. *compared to T1. **compared to T2. ***compared to T2 and T1.

**Figure 4:**
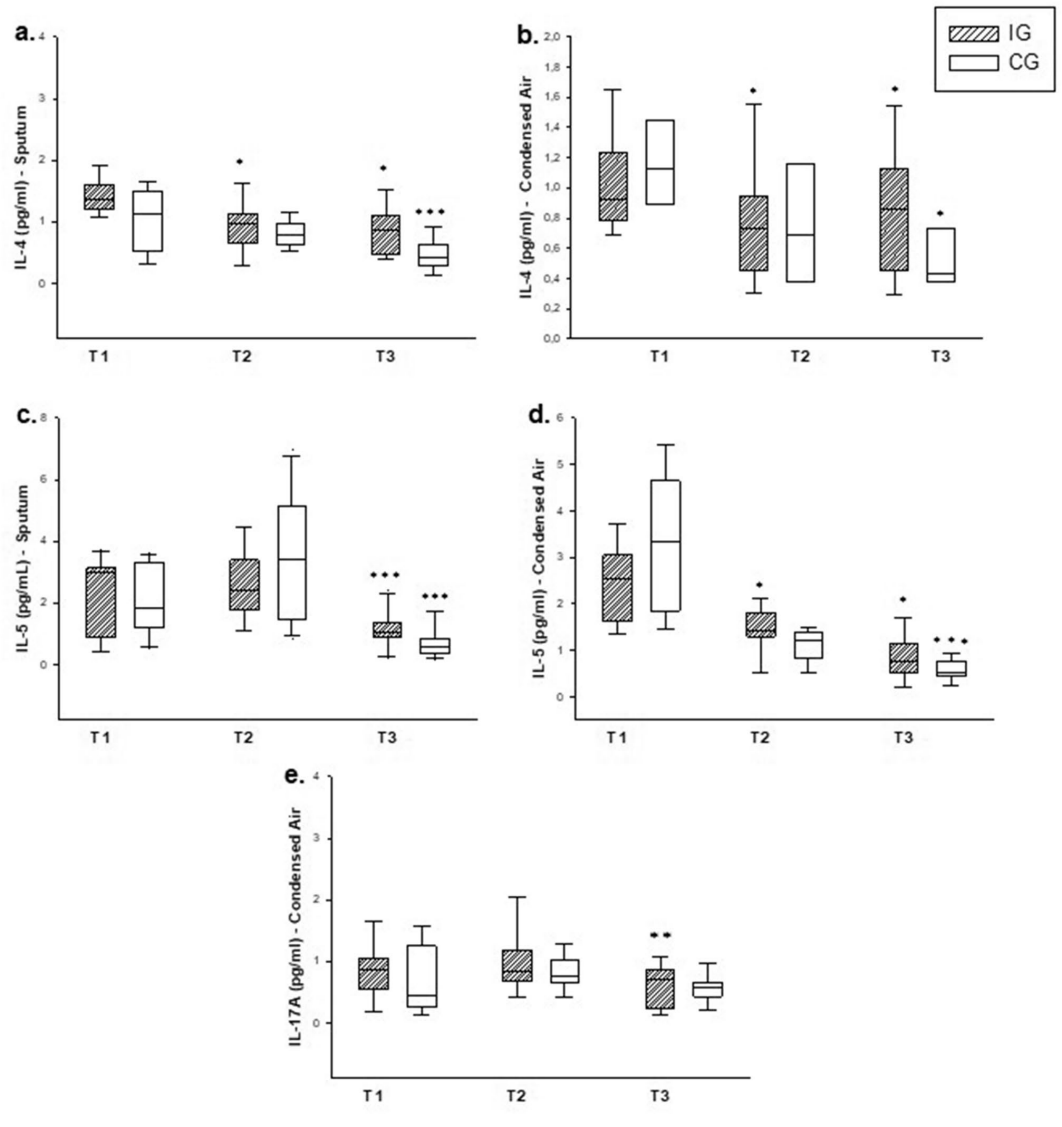
Total cytokine count in the three evaluations (T1, T2 and T3). IG = intervention group. CG = control group. T1 = day 0 (baseline). T2 = day 28. T3 = day 56. EBC = exhaled breath condensate. IS = induced sputum. (**a**, **b**) IL-4 and IL-5 in IS. (**c**, **d**) IL-4 and IL-5 in EBC. (**e**) IL-17A in EBC. *Compared to T1. **Compared to T2. ***Compared to T2 and T1. The boxes represent the 25th to 75th percentiles (SD), the lines inside the boxes represent the median values, and the bars represent the 10th and 90th percentiles. Statistical analysis: One-way ANOVA (intragroup analysis).

In cytokines analysis, regarding the IG, there was a decrease in expression of IL-4 in T2 and T3 compared to T1 in IS supernatant (*p < 0.001) (Fig. 4a) and EBC (*p < 0.001) (Fig. 4b) (Table 3). Moreover, there was a decrease in IL-5 levels in IS at T3 compared to T2 and T1 (p < 0.01) (Fig. 4c) and in EBC compared T2 and T3 with T1 (p < 0.001) (Fig. 4d) (Table 3).

In CG, we observed decreased expression of IL-4 values at T3 compared to T2 and T1 in IS (*p = 0.001) (Fig. 4a) and at EBC analysis we found a decrease at T3 compared to T1 (*p = 0.011) (Fig. 4b). Regarding to IL-5 measurement, the CG presented decreased values at T3 compared do T2 and T1 in the IS (*p < 0.001) and also in the EBC (*p < 0.001) (Fig. 4c) (Table 3).

Regarding the EBC analysis of interleukin IL-17A, there was a decrease in the IG group in the T3 evaluation when compared with T2 (Fig. 4e, Table 3) (*p = 0.028). In IS supernatant, it was not possible to analyze the IL-17 data in either group since the values were lower than the detection curve.

### Effects of the educational intervention—odds ratio

The effect of the educational intervention was compared between the groups (IG and CG) through the odds ratio and chi-square test among the 3 study visits (T1, T2, T3), including clinical, functional and inflammatory parameters (Table 4).

**Table 4:** Odds ratios of the main outcomes among study’s measurements dates.

Outcome	Time interval	Odds ratio (IG/CG)	95% Confidence interval	Between group (p value)
Eosinophils (%)	T1–T2	3.300	1.060–10.482	0.036
	T2–T3	5.556	1.742–17.714	0.003
	T1–T3	5.400	1.627–17.921	0.004
NO (ppb)	T1–T2			
	T2–T3	2.000	0.716–5.590	0.184
	T1–T3	0.699	0.250–1.949	0.492
IL-4 (sputum)	T1–T2	9.143	0.859–97.265	0.062
	T2–T3	0.198	0.033–1.200	0.115
	T1–T3		0.913–1.326	0.393
IL-5 (sputum)	T1–T2	2.857	0.405–20.141	0.371
	T2–T3	0.400	0.068–2.337	0.400
	T1–T3	0.800	0.149–4.297	1.000
IL-4 (EBC)	T1–T2	1.167	0.862–1.579	0.318
	T2–T3	0.083	0.068–2.337	0.063
	T1–T3	0.733	0.149–4.297	0.263
IL-5 (EBC)	T1–T2	0.286	0.022–3.669	0.543
	T2–T3			
	T1–T3	0.800	0.587–0.995	0.150
IL-17A (EBC)	T1–T2	0.917	0.256–3.286	0.894
	T2–T3	0.405		0.432
	T1–T3	1.778	0.471–6.711	0.394
FEV_1_ before BD (%)	T1–T2	2.556	0.861–7.590	0.088
	T2–T3	0.600	0.210–1.715	0.339
	T1–T3	4.156	1.382–12.493	0.010
FEV_1_ after BD (%)	T1–T2	5.714	1.806–18.080	0.002
	T2–T3	0.960	0.331–2.788	0.940
	T1–T3	3.055	1.042–8.953	0.039
AQLQ	T1–T2	1.625	0.554–4.762	0.375
	T2–T3	0.917	0.331–2.538	0.867
	T1–T3	1.297	0.462–3.646	0.621
BDI (depression)	T1–T2	1.203	0.411–3.525	0.736
	T2–T3	0.446	0.159–1.252	0.123
	T1–T3	0.889	0.301–2.626	0.831
ACT	T1–T2	1.131	0.393–3.254	0.819
	T2–T3	0.542	0.190–1.543	0.249
	T1–T3	0.587	0.209–1.648	0.311
ACQ	T1–T2	1.847	0.642–5.315	0.253
	T2–T3	0.961	0.337–2.736	0.940
	T1–T3	3.911	1.293–11.383	0.014

*IG* intervention group, *CG* control group, *T1* day 0 (baseline), *T2* day 28, *T3* day 56, *EBC* Exhaled Breath Condensate, *BD* bronchodilator, *FEV*_*1*_ Forced Expiratory Volume in one Second, *AQLQ* Asthma Quality Life Questionnaire, *BDI* Beck Depression Inventory, *ACT* Asthma Control Test, *ACQ* Asthma Control Questionnaire.

A significant difference was observed in the percentage of: eosinophils in IS between T1 and T2 (1.060–10.482, OR 3.300, p = 0.036), T2–T3 (1.742–17.714, OR 5.556, p = 0.003) and T1–T3 (1.627–17.921, OR 5.400, p = 0.004); FEV_1_ before BD among T1–T3 (1.382–12.493, OR 4.156, p = 0.010); FEV_1_ after BD among T1–T2 (1.806–18.080, OR 5.714, p = 0.002) and T1–T3 (1.042–8.953, OR 3.055, p = 0.039); ACQ between T1 and T3 (1.293–11.383, OR 3.911, p = 0.014) (Table 4).

## Discussion

In the present study, we found that asthmatic patients with the disease partly controlled and uncontrolled who underwent an educational intervention showed improved clinical control of asthma, pulmonary function, markers of airway inflammation, and quality of life and decreased levels of depression.

We showed the importance of an educational activity for a short period of time. This has led to a better lung function, decreased inflammation and levels of depression, and a better quality of life. This improvement occurred even without changing the previous pharmacological treatment of the patients, thus showing the importance of the educational approach in the management of asthma.

An effective guided asthma self-management education may help patients and reduce morbidity^2,32,33^, asthma-related hospitalizations, emergency department visits and unscheduled doctor or clinic visits, missed work/school days, and night awakening^2,34,35^. The use of inhalation devices promotes a high concentration of medication in the airways, more rapid onset of action, and fewer systemic adverse effects than systemic delivery. For this to happen, the correct use of the inhalation device must be learned and maintained. In this context, skills training to use inhaler devices effectively are essential in an individualized self-care plan for asthma patients^2^.

Some previous studies have shown that educational activities and self-management related to asthma features have been associated with better control of the disease in both adults and children^2,9,31,36^. Boulet et al. showed significant improvements after an educational program, such as a reduction in unscheduled visits, inappropriate use of medications and an increase in FEV_1_ in mild to moderate asthmatic patients after 1 year of follow-up^37^. In another study, asthmatic and COPD patients showed better clinical control and lung function after submitting to a 6–8 follow-up educational program^38^. In our study, we also found an increased FEV_1_ after one (before and after bronchodilator) and 2 months (after bronchodilator) of educational intervention follow-up, that is, the improvement of this parameter after the educational activity.

Lv et al. compared an SMS with a traditional educational program and an outpatient ordinary service (control group)^39^. They found that both groups that received an educational program presented increased scores on perceived control asthma questionnaires, although scores in the SMS group were higher than those in the traditional educational program^39^. Continuing medical program education in asthma care was shown to improve parent-reported provider communication skills, the number of days affected by asthma symptoms, and asthma health care use^40^.

Other authors have evaluated quality of life as an auxiliary measure of asthma treatment and its control^27,41–43^ and the negative effects of uncontrolled asthma on patients' quality of life^44^. In our study, asthmatic patients from IG presented better quality of life after 1 and 2 months (T2 and T3) of protocol intervention. Janson et al.^36^ showed that individualized asthma self-management education resulted in a decrease in nighttime awakenings, improved perceived control of asthma, better adherence to treatment, and improved clinical parameters of the disease and quality of life.

However, we did not observe differences when comparing ACT and ACQ scores in either group. In the same way, Maricoto et al.^38^ also did not find differences between groups after an educational intervention regarding proper use of ICS in ACT scores. França-Pinto et al. did not show a difference in ACQ after an aerobic training program of asthmatic patients, although they presented a better quality of life and better scores of depression^27^. However, when the effect of the educational intervention was compared between the groups (IG and CG) through the odds ratio and chi-square test among the 3 study visits (T1, T2, T3) we observed a significant difference in the percentage of ACQ between T1 and T3.

One strength of our study is that even the CG presented more cough-free days and better IL-4, IL-5, and depression levels after the protocol. We hypothesized that these positive results could be motivated by the use of peak flow to fill a diary of symptoms and by some calls received from a health professional; thus, it was enough to help them feel self-confident to better manage the disease.

Depression is an important asthma comorbidity and has been associated with worsening disease control in adults^45,46^. Plourde et al.^47^ validated the BDI, one of the most widely used questionnaires to screen depression in health research areas^24,47–50^, to be used in asthmatic adult patients.

We found better depression scores in the IG after 1 month (T2) of educational intervention and after 2 months (T3) in the CG. When invited to participate in a survey, the participant’s attention is focused on the subject in question, that is, to have more health care. It is possible that the fact of having applied questionnaires about your illness, quality of life, depression, may have influenced further reflection and self-care, generating some positive results even in patients in the control group.

Stoop et al. carried out a study with people with diabetes, asthma or COPD and the subjects answered questionnaires after 3, 6, 9, 12 and 18 months. The intervention group received a 12-month stepped care treatment and monitoring of symptoms. The control group received usual care plus monitoring by filling in a postal questionnaire every 3 months. Eighteen months post screening, the difference in symptoms of depression between intervention and control group was not significant anymore^51^.

Regarding inflammatory parameters, the exact role of FeNO in asthma is still not clear^2,52^. Some studies have associated FeNO with eosinophilia in asthmatic patients^2,53,54^. However, according to GINA^2^, this measure has not been established as useful for ruling in or ruling out a diagnosis of asthma; among other factors, it is not elevated in some asthma phenotypes, such as neutrophilic asthma^3,55^. The majority of our patients had paucigranulocytic asthma, which may explain the decreased values of FeNO after educational intervention (Table 2).

Eosinophils have an important role in the immunological response in asthma. Studies have demonstrated a relationship between eosinophils and airway hyperresponsivity and remodeling as well as its relationship with asthma severity^30,56^. A retrospective study of asthmatic adult patients followed up for 2 years showed a relationship between better disease control and a decrease in the number of eosinophils. They suggested that the control of asthma could be a consequence of intensifying treatment with ICS^57^. In our study, patients who were submitted to the educational program presented a decrease in the number of eosinophils in the sputum, which may be due to better adherence to ICS.

Another mediator related to eosinophils and the inflammatory process of asthma is Th2 cytokines, such as IL-4 and IL-5^1,30,38,58^. The ERS/ATS Task Force (2020) made recommendations on the use of novel therapies for severe asthma, specifically biologics for type 2 high asthma, such as the anti-IL-5 mepolizumab and reslizumab, the IL-5 receptor antagonist benralizumab and dupilumab, the IL-4 and IL-13 α-chain receptor antagonist. Those biologics showed efficacy for severe uncontrolled eosinophilic asthma phenotypes, particularly for those with severe corticosteroid-dependent asthma^59^. Thus, decreasing the levels of these cytokines with only a short-term educational intervention can bring benefits to patients.

In the IG group, it has already been possible to observe with one month of educational intervention an improvement of inflammation, seen through the reduction of IL-4 and IL-5, both as evaluated by IS and EBC; this reduction was also observed after 2 months of intervention. In the CG, although there has been a decrease of these parameters, this reduction only occurred after 2 months. CG presented a decrease in these cytokines but they did not present improvement in other parameters that could represent disease control, such as lung function, the number of eosinophils and quality of life. Although these cytokines are involved in asthma pathogenesis, other mediators are also important to achieve the control of asthma symptoms.

Although allergic asthmatic features are linked to Th2 cytokines, recent studies have associated Th2 with Th17 cytokines. Bullens et al. found an increase in the expression of IL-17 mRNA in the sputum of asthmatic patients compared to healthy controls^60^. Barczyk et al. also presented a correlation of increased levels of IL-17 in asthmatic patients with bronchial hyperreactivity^61^. Camargo et al. showed that therapy with anti-IL-17 could be used to control the inflammatory process in an exacerbated asthma model^62^. In addition, Fattahi et al. found that lower levels of IL-17 + cells in asthmatic patients were associated with atopy and ICS use^63^. We observed decreased expression of IL-17A at EBC only in IG, suggesting a possible pathway to explain reduced inflammation in these patients.

We believe that one of the limiting factors of this study may have been the use of dithiothreitol (DTT) for processing sputum supernatant. It is indicated for better preparation of slides^64^; however, its use has already been described as potentially harmful for differential cell analysis^65,66^. This procedure may have influenced the analysis of cytokines, a fact that was observed only after the freezing of samples. Another factor to be considered is the sample size, which could be expanded in future studies. It should also be considered that the CG may have influenced some of the results despite not having received the educational intervention in the first two visits. The fact that CG participated in the research may have influenced the results in a beneficial way and may have inferred some of the results. Despite these limitations, our study has several strengths. We showed that an educational intervention performed for a short period of time, applied by a trained professional, can produce benefits to patients in relation to inflammation, lung function, quality of life and depression levels.

In summary, we showed that an educational program directed to asthmatic patients in addition to physician care has an important role in disease control. Identifying patients’ needs, beliefs and behaviors can indicate where improvements should be focused to help people and plan future interventions. In this study, this benefit was evidenced by a reduction in airway inflammatory markers, such as IL-4, IL-5, IL-17A, FeNO and lung function, which consequently resulted in better quality of life and decreased depression levels. Finally, we suggest that health educational programs should be part of asthma management.

## Data Availability

All relevant data are within the paper and; the main data can also be accessed at: https://clinicaltrials.gov/ct2/show/results/NCT03655392. Clinical Trial Registration: ClinicalTrials.gov; number: NCT03655392; URL: www.clinicaltrials.gov. It was first registered in 03/28/2018 (retrospectively recorded). Any additional data can be requested from the corresponding author or the last author.
